# Computerized Eye-Tracking Training Improves the Saccadic Eye Movements of Children with Attention-Deficit/Hyperactivity Disorder

**DOI:** 10.3390/brainsci10121016

**Published:** 2020-12-21

**Authors:** Tsz Lok Lee, Michael K. Yeung, Sophia L. Sze, Agnes S. Chan

**Affiliations:** 1 Department of Psychology, The Chinese University of Hong Kong, Shatin, New Territories, Hong Kong, China; tllee@link.cuhk.edu.hk (T.L.L.); lmsze@cuhk.edu.hk (S.L.S.); 2 Department of Rehabilitation Sciences, The Hong Kong Polytechnic University, Hong Kong, China; kin-chung-michael.yeung@polyu.edu.hk; 3 Research Center for Neuropsychological Well-Being, The Chinese University of Hong Kong, Shatin, New Territories, Hong Kong, China

**Keywords:** eye-tracking, saccade, fixation, cognitive training, ADHD

## Abstract

Abnormal saccadic eye movements, such as longer anti-saccade latency and lower pro-saccade accuracy, are common in children with attention-deficit/hyperactivity disorder (ADHD). The present study aimed to investigate the effectiveness of computerized eye-tracking training on improving saccadic eye movements in children with ADHD. Eighteen children with ADHD (mean age = 8.8 years, 10 males) were recruited and assigned to either the experimental (*n* = 9) or control group (*n* = 9). The experimental group underwent an accumulated 240 min of eye-tracking training within two weeks, whereas the control group engaged in web game playing for the same amount of time. Saccadic performances were assessed using the anti- and pro-saccade tasks before and after training. Compared to the baseline, only the children who underwent the eye-tracking training showed significant improvements in saccade latency and accuracy in the anti- and pro-saccade tasks, respectively. In contrast, the control group exhibited no significant changes. These preliminary findings support the use of eye-tracking training as a safe non-pharmacological intervention for improving the saccadic eye movements of children with ADHD.

## 1. Introduction

Saccadic control is important in daily life since saccades form the basis of visual searching in a complex environment, allowing oneself to direct attention to information that is relevant to one’s goal while at the same time excluding irrelevant information [1,2,3]. As such, effective and efficient saccadic abilities facilitate the goal-directed acquisition of information from the external world. While pro-saccades underlie the orienting to relevant information, anti-saccades, which draw on interference control abilities [4], underlie the filtering of irrelevant information. Also, saccadic movements have been shown to relate to other cognitive abilities, such as intelligence and working memory [5].

While inattention and hyperactivity are two hallmark features of attention-deficit/hyperactivity disorder (ADHD), eye movement abnormalities are commonly seen in individuals with this disorder. For instance, some studies have shown that compared to typically developing children, children with ADHD showed significantly longer saccade latency in an anti-saccade task [6,7]. Similarly, in the pro-saccade task, children with ADHD showed poorer saccade accuracy [6,8]. These results suggest that children with ADHD have difficulty in suppressing unwanted saccades and controlling their eye fixations voluntarily. It was also found that eye movement abnormalities were positively correlated with the severity of the ADHD symptoms [9]. Therefore, interventions related to the eye movement abnormalities of children with ADHD are of clinical importance.

Notwithstanding research on the eye movement abnormalities among children with ADHD, relatively little research has focused on interventions to improve their eye gazing abilities. Some studies have found that pharmacological interventions, such as methylphenidate administration, improved both the pro- and anti-saccades of children with ADHD [10,11]. However, medication may cause side effects such as insomnia, decreased appetite, and headaches [12]. Thus, non-pharmacological treatments may be an alternative intervention. Recently, one study has investigated the therapeutic benefits of an eye-tracking training game on visual attention [13]. In the training, participants with ADHD caught snowflakes that appeared on the screen when no fire was present and inhibited movement when the fire was present. Participants’ fixation gaze control was assessed using the “frog task”, where participants first fixated at the central target (i.e., a frog) and then waited upon the appearance of a tadpole or a fish on one side of the screen, pressing a button when the tadpole appeared but refraining from pressing the button when the fish appeared. Fixation gaze control was operationalized as the number and duration of fixations at the central frog (i.e., the ability to make stable fixations). After nine training sessions within three weeks, 14 participants in the experimental group, aged 8 to 15 years, showed a significant improvement in fixation gaze control in terms of fewer and longer fixations on the central frog during the task. On the contrary, participants in the control group, who played the game using the mouse, did not show any significant changes. These results suggested possible therapeutic effects when combining visual training with the eye-tracking technique. However, it remains unclear whether computerized eye-tracking training can improve goal-directed saccadic eye movements, such as the ability to suppress unwanted saccades, in children with ADHD.

Our team has developed a computerized eye-tracking training program and clinically applied it over several years. This program consists of different eye-tracking tasks, which require participants to search for some targets and fixate at them, ignore distractions when they appear, and closely follow some moving targets. This program was developed based on the association between eye movement control and frontal lobe functioning [14,15]. Specifically, saccadic eye movements are associated with attention [16] and flexible thinking [17], and anti-saccadic eye movements additionally draw on inhibitory control. These processes are mainly mediated by the dorsolateral prefrontal cortex and dorsal anterior cingulate cortex [18,19]. Since frontal lobe dysfunction is common in ADHD [20,21,22], our training program was designed to train children with ADHD’s frontal cognitive function via improving their saccadic eye movement control. We have observed encouraging results in the clinic; that is, children have demonstrated significant behavioral improvements in attention, inhibition, and mental flexibility after the training.

Indeed, the effectiveness of our training could be evaluated by investigating its effects on eye movement control and frontal cognitive function. We have recently examined the effect of this training program in a group research setting and found behavioral improvements in inhibitory control across computerized and paper-and-pencil neuropsychological tasks in primary school children with ADHD [23]. To elucidate the physiological mechanism underlying the change in cognitive function, the present study examined the effect of this computerized eye-tracking training program on saccadic eye movements in children with ADHD using the classic pro- and anti-saccade tasks, which measured goal-directed saccadic initiation and suppression, respectively. Throughout the training the children were trained on their fixation and visual searching skills, since they needed to search for the targets and ignore the distractions at the same time during the training tasks. Therefore, it was hypothesized that the eye-tracking training improved the participants’ eye movement.

## 2. Materials and Methods

### 2.1. Participants

Eighteen participants who had been diagnosed with ADHD by a registered clinical psychologist or a psychiatrist were recruited through online advertisement. The selection requirements included: (1) aged between 6 and 12 years, (2) was attending mainstream primary school, (3) with normal or corrected-to-normal vision, and (4) with parent-reported behavioral and attentional problems. All participants had an intelligence quotient (IQ) > 70, estimated using the short form of the Wechsler Intelligence Scale for Children–Fourth Edition (Hong Kong) (WISC-IV-HK:SF [24]). It has been suggested that the inclusion of children with ADHD with low IQ scores (between 70 to 85) may better represent the ADHD population [25]. Therefore, a lower cut-off was used in this study. Participants were assigned to either the experimental or the control group, with nine children in each group.

### 2.2. Procedure

Before training, participants’ IQ was assessed using the WISC-IV-HK:SF [24]. Parents or guardians of the participants were asked to complete a questionnaire on the child’s family background, medical history, and ADHD symptoms using Conners’ Parent Rating Scale-Revised: Short Form (CPRS-R:S [26]). The experimental group underwent the eye-tracking training, with eight training sessions within two weeks. Each session consisted of three 10-minute training tasks, with a 5-minute break between the training tasks. For the control group, children played some common web computer games (i.e., Tetris, Pacman, Zoo Keeper) for the same amount of time that the experiment group spent in training. The post-assessment, which followed the same protocol as the pre-assessment, was conducted to both the experimental and control groups two weeks after the pre-assessment.

This experiment was also approved by the Joint Chinese University of Hong Kong–New Territories East Cluster Clinical Research Ethics Committee (Code: NTEC-2018-0298). Informed consents were obtained from all participants and their parents or legal guardians.

### 2.3. Tests and Materials

An anti-saccade task and a pro-saccade task were used to assess goal-directed saccadic eye movements. These tasks were run using a 23″ screen (screen resolution: 1920 × 1080) and programmed using PsychoPy3 [27]. Eye movements were recorded with a 150-Hz eye tracker (Gazepoint GP3 HD, Gazepoint Research Inc., Vancouver, Canada). Participants successfully underwent a nine-point calibration before the tasks started.

The anti-saccade task was employed to assess the participants’ inhibitory control [7,28]. During the test, participants sat 60 cm in front of the computer monitor with their heads positioned on a chin rest to avoid unnecessary head movements and to maintain the viewing distance. Each trial began with a fixation point shown at the center of the monitor for 800 ms. During this period, participants were instructed to keep their eyes fixating at this point. The fixation point then disappeared for 200 ms, and a target cue was shown on either the left or right side of the central fixation point with a subtended visual angle of 11.3° for 1000 ms. Participants were asked to fixate at the opposite direction of the target cue with the same amplitude from the original central fixation point as fast and accurately as possible. The trial ended with a 1000 ms blank period. The anti-saccade task consisted of 40 trials, with 20 left-sided and 20 right-sided cues presented in a pseudorandomized order. A pro-saccade task was also employed to assess the participants’ attention. The monitor configuration, equipment, and task settings were the same as in the anti-saccade task; however, in this task, participants were instructed to fixate on the target cue when it appeared.

### 2.4. Computerized Eye-Tracking Training

The training was conducted with a Tobii Eye Tracker 4C (Tobii, Stockholm, Sweden), a 23” external monitor, and a desktop computer running Windows 7. The eye tracker was a non-contact tracker with a temporal resolution of 90 Hz and head tracking. Participants sat approximately 50 cm in front of the monitor, on which the eye tracker was placed. After a six-point calibration, participants engaged in the training. The computer training program was developed and licensed by the Pro-talent Association Ltd (a non-profit organization) in Hong Kong. This program was developed based on scientific evidence regarding eye gazing and frontal lobe processing with the purpose of improving inhibitory control, mental flexibility, and attention. The training program consisted of six modules, and each module had three levels. The goal was the same across modules—to improve attention and impulse control—but the way to play the games varied. In each game, the participants were required to fixate on a target, and a score was given once they had fixated for long enough. For example, in one game, the participants had to fixate a falling stone for long enough before it fell down. The fixation duration required to obtain the score increased as the level advanced. The training time of each module was 10 min, and the participants could choose to continue or quit after each module. The program automatically moved to the next level when the child obtained a score of 80 or higher. In each training session, the children were asked to fixate their eye gaze on a target and ignore distractions. 

### 2.5. Data Analysis

**Figure 1:**
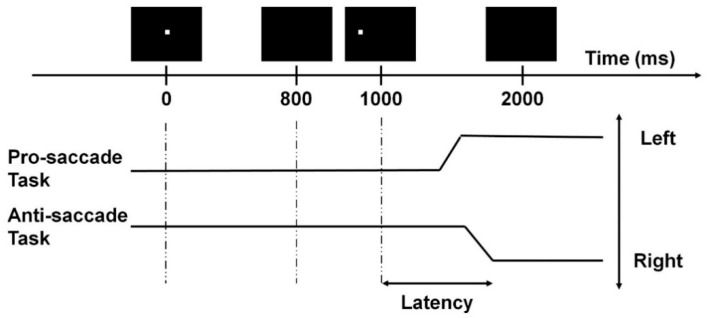
Example of a saccade trial with a target cue appearing on the left. The two black solid lines represent the correct saccadic responses.

The saccade latency and accuracy were evaluated for both the anti- and pro-saccade tasks (Figure 1). All fixation and saccadic events were detected by the inbuilt Gazepoint analysis software. First, all the gaze points with detected eye blinks were removed. Then, the first saccade that happened 50 ms after the target cue’s onset with a 4° amplitude was identified for each trial [7]. A trial was classified as correct when the first saccade was in the correct direction (i.e., towards the target cue in the pro-saccade task, and away from the target cue in the anti-saccade task). Then, accuracy was calculated by dividing the total number of correct trials by the total number of trials (i.e., 40). The mean saccade latency was computed by averaging the reaction time of the first saccades in correct trials. Saccade accuracy was the percentage of trials with correct saccades. A total of 2422 (84.1%) trials were valid and included in analysis. All the above computations were done using Matlab^®^ R2019a (The MathWorks, Natick, MA, USA).

To analyze the data, first, independent sample *t*-tests and chi-squared tests were used to compare the demographical, intellectual, and clinical characteristics between the experimental and control groups. Next, mixed ANOVA were performed to evaluate the time (pre, post) × group (experimental, control) interactions. Paired *t*-tests were performed to compare the task performance before and after training in order to evaluate the treatment effect. Cohen’s d was calculated to examine the effect size. All statistical analyses were performed using SPSS 24.0 software (IBM Corporation, Armonk, NY, USA), and the significance level was set at 0.05 for all tests (two-tailed).

## 3. Results

### 3.1. Demographic and Baseline Information

Table 1 presents the demographical, intellectual, and clinical characteristics of the children with ADHD in the experimental and control groups. In the pre-assessment, there were no significant differences in terms of demographic variables, ADHD symptoms, IQ, and test performance between the experimental and control groups (*p*s from 0.24 to 0.93).

### 3.2. Improvement in the Anti-Saccade Task after Training

The changes in saccade performance are also presented in Table 2. For the anti-saccade task, while none of the time × group interaction effects were significant (*F*(1,25) = 0.26–1.65, *p* = 0.22–0.62, $\eta_{p}^{2}$ = 0.02–0.09)), a medium-to-large effect was observed for the saccade latency ($\eta_{p}^{2}$ = 0.09). Paired *t*-tests showed that the experimental group had a significantly shorter saccade latency after training compared to the baseline (*t*(8) = 2.63, *p* = 0.030, *d* = 0.87), with a large effect size of difference. In contrast, the control group did not show a significant change (*t*(8) = 0.32, *p* = 0.76, *d* = 0.11), and the effect size was very small, if not negligible. Neither group showed significant changes in the saccade accuracy (*p*s > 0.10). The above results suggest that only the experimental group showed significant improvement in anti-saccades after intervention.

### 3.3. Improvement in the Pro-Saccade Task after Training

The saccade performance in the pro-saccade task was then analyzed. None of the time × group interaction effects were significant (*F*(1,25) = 0.26–0.56, *p* = 0.46–0.61, $\eta_{p}^{2}$ = 0.02–0.03). However, while neither group showed significant improvement in saccade latency, the experimental group showed a significant increase in saccade accuracy (*t*(8) = 2.60, *p* = 0.032, *d* = 0.87), which was of large effect size and not observed in the control group (*p* = 0.10). The above results suggested that the computerized eye-tracking training also benefited ADHD participants’ pro-saccades.

## 4. Discussion

This study examined the effectiveness of computerized eye-tracking training on saccadic eye movements in children with ADHD. After 240 min of training, only the experimental group exhibited a significant decrease in saccade latency in the anti-saccade task and a significant increase in saccade accuracy in the pro-saccade task. No children self-reported side-effects throughout the training program. Given that eye movement deficits are present in children with ADHD [6,7,8], the present study suggested the therapeutic possibility of computerized eye-tracking training to improve saccadic eye movements in children with ADHD.

Our findings contribute to the literature by extending a previous eye-tracking training study [13], in which improved fixation gaze control was found in children with ADHD after 540 min of visual attention training that spanned three weeks. Compared to that study, the present study had a shorter total training time (540 min vs. 240 min). In addition to searching for static targets and resisting static distractors, the present training also trained children’s gaze pursuing abilities since they were asked to track some moving objects. Furthermore, in García-Baos et al. [13], the group × time interaction had a $\eta_{p}^{2}$ of 0.006 for the number of fixations and a $\eta_{p}^{2}$ of 0.01 for the duration of fixations. Compared to the effect sizes of our study ($\eta_{p}^{2}$ = 0.02–0.09), the present training seems to yield stronger effects. The present study also explored the training effect in children of a younger age using traditional saccade test paradigms. Since only a short training time (i.e., around 240 min) was enough to induce significant positive effects, our findings suggest that cognitive training using the eye-tracking technique may be a cost-effective intervention for children with ADHD.

During the intervention, children were asked to fixate on targets while ignoring distractions. This trained their ability to voluntarily control their eye movements, which was associated with their frontal lobe functioning [14]. Regions of the frontal lobes, such as the frontal eye fields, are responsible for saccade initiation [29]. In addition, the supplementary eye fields and the dorsolateral prefrontal cortex also play important roles in the regulation [30] and executive control [31] of saccade, respectively. It is speculated that the functioning of multiple sub-regions within the frontal lobes was strengthened after the training, resulting in improved saccadic control.

We recently found significant behavioral improvements across neuropsychological and experimental tasks that probed frontal lobe function (i.e., inhibitory control and mental flexibility) in children with ADHD after 240 min of eye-tracking training [23]. The present findings extend the previous findings by demonstrating the change in goal-directed saccadic eye movements following eye-tracking training. Notably, the participants with ADHD in the present study did not participate in the other study, and both samples had the same age range (i.e., 6–12 years). The training times were also identical. Thus, the present study provides preliminary evidence that eye-tracking training may influence children’s executive function by improving their eye movement control, reinforcing the benefits of incorporating cognitive training with the eye-tracking technique.

The present training primarily improved latency in the anti-saccade task and accuracy in the pro-saccade task. While latency reflects the efficiency of information processing, accuracy reflects the decision-making process. Accordingly, we speculate that these different processes may be improved by the present computerized training in a context-dependent manner. As such, our training might improve the decision-making process during orienting to relevant targets and the efficiency of processing and solving conflicts while suppressing unwanted saccadic movement.

The present study examined the immediate effect of eye-tracking training, and follow-up studies should be conducted to determine whether the training can produce a long-lasting effect, and whether the training effect can be transferred to functions in daily life. Still, the present study provides some preliminary evidence that 240 min of computerized eye-tracking training can potentially improve children with ADHD’s saccadic eye movements. These findings highlighted the incorporation of eye-tracking in cognitive training, and computerized eye-tracking training may serve as one of the safe non-pharmacological interventions for ADHD. Further studies can be done to examine whether the effectiveness of computerized eye-tracking training are generalizable to children with other neurodevelopmental disorders, such as autism spectrum disorders and dyslexia, who also exhibit abnormal saccadic control [32,33].

## Figures and Tables

**Table 1:** Demographical, intellectual, and clinical characteristics of the children with ADHD in the experimental (*n* = 9) and control groups (*n* = 9). CPRS-R:S = Conners’ Rating Scale for Parents-Revised: Short Form; WISC-IV-HK:SF = Short form of Wechsler Intelligence Scale for Children—Fourth Edition (Hong Kong).

Variables	Control (*n* = 9)		Experimental (*n* = 9)				
	*M*	*SD*	*M*	*SD*	*t/χ^2^*	*p*	*d/V*
Age (years)	8.4	1.2	9.1	1.3	1.22	0.24	0.57
Gender (F/M) ^a^	5/4		3/6		0.9	0.34	0.06
**CPRS-R:S**							
ADHD index	23.7	8.3	24	8.6	0.08	0.93	0.04
**WISC-IV-HK:SF**							
Estimated IQ	89.6	12.8	90.4	14.9	0.14	0.89	0.06

^a^ Chi-squared tests were used to compare groups.

**Table 2:** Changes in task performance before and after the eye-tracking training in the experimental (*n* = 9) and control (*n* = 9) groups.

Measures	Control (*n* = 9)					Experimental (*n* = 9)				
	Pre	Post	*t*	*p*	*d*	Pre	Post	*t*	*p*	*d*
	*M (SD)*	*M (SD)*				*M (SD)*	*M (SD)*			
**Anti-saccade task**										
Saccade latency (ms)	517.8 (80.5)	526.9 (90.9)	0.32	0.76	0.11	537.3 (43.5)	506.6 (51.3)	2.62	0.030 *	0.87
Saccade accuracy (%)	49.4 (27.1)	60.8 (28.4)	1.88	0.10	0.63	66.4 (22.8)	73.6 (20.7)	1.33	0.22	0.44
**Pro-saccade task**										
Saccade latency (ms)	564.5 (82.6)	516.0 (44.5)	1.89	0.10	0.63	578.7 (76.5)	557.9 (58.8)	0.79	0.45	0.26
Saccade accuracy (%)	66.9 (15.8)	77.8 (10.5)	1.86	0.10	0.62	72.2 (9.5)	79.7 (7.3)	2.60	0.032 *	0.87

* *p* < 0.05.
